# Evaluation Soybean Cultivars for Reaction to *Heterodera glycines* Populations HG Types 7 and 1.3.4.7 in Northeast China

**DOI:** 10.3390/life13010248

**Published:** 2023-01-16

**Authors:** Jingsheng Chen, Yuanyuan Zhou, Yanfeng Hu, Di Zhao, Changjun Zhou, Rujie Shi, Miao Sun, Li Zhang, Guowei Chen, Haiyan Li, Lijie Chen, Guosheng Xiao

**Affiliations:** 1 College of Biology and Food Engineering, Chongqing Three Gorges University, Chongqing 404000, China; 2 College of Agronomy, Heilongjiang Bayi Agricultural University, Daqing 163000, China; 3 Key Laboratory of Mollisols Agroecology, Northeast Institute of Geography and Agroecology, Chinese Academy of Sciences, Harbin 150081, China; 4 Nematology Institute of Northern China, Shenyang Agricultural University, Shenyang 110866, China; 5 Daqing Branch, Chinese Academy of Sciences of Heilongjiang Province, Daqing 163316, China

**Keywords:** *Glycine max*, *Heterodera glycines*, HG type, resistance screening, soybean

## Abstract

Soybean cyst nematode *Heterodera glycines* (SCN) is a major threat to global soybean production. Effective management of this disease is dependent on the development of resistant cultivars. Two SCN HG Types, 7 and 1.3.4.7. were previously identified as prevalent *H. glycines* populations in Northeast China. In order to evaluate soybean cultivars resistant to local SCN populations, 110 domestic commercial soybeans from different regions of Northeast China were assessed in the greenhouse to determine their potential as novel sources of resistance. The results suggested that cultivars responded differently to the two HG types. Of the 110 soybean cultivars evaluated, 24 accessions were classified as resistant or moderately resistant to HG Type 7, and five cultivars were classified as resistant or moderately resistant to HG Type 1.3.4.7. Among the tested cultivars, Kangxian 12 and Qingdou 13 had resistance response to both HG types 7 and 1.3.4.7. Thus, these broad-based SCN cultivars will be the valuable materials in the SCN resistance breeding program.

## 1. Introduction

Soybean cyst nematode continues to be the most crucial thread to soybean (*Glycine max* (L.) Merr.) production worldwide. In 1899, China reported the first occurrence of “fire-burned seedlings” caused by SCN in western Heilongjiang Province [1]. It was classified as *Heterodera glycines* by Ichinohe until 1952 [2]. Nowadays, it spread to most soybean planting-areas around 22 provinces of China [3,4,5,6], which caused an annual yield loss of more than 120 million dollars [7].

SCN has been managed through rotation with nonhost crops, SCN-resistant soybean cultivars, bio-control and nematicide applications, and so on [8,9]. Currently, genetic resistance is the most economical, effective, and environmentally sustainable management means to control this nematode [10,11]. For soybean growers, the development of SCN resistant cultivars has been a significant achievement. Pickett is the first SCN-resistant cultivar through three backcrosses between Peking and the susceptible cultivar Lee [12]. Usually, the yield of resistant cultivars was substantially higher than that of susceptible cultivars in fields with SCN infestation [13,14].

Heilongjiang Province is the major soybean producing region in China. The northern part of this province is the largest planting area of soybeans. More than one-third of total soybean production in China is located in this area, where SCN caused significant yield reductions in soybean producing regions [15]. Eleven *H. glycines* (HG) Types 0, 1.2.3.5.7, 1.2.3.7, 1.3.4.7, 1.3.7, 2, 2.5.7, 2.7, 6, 6.7, and 7, were reported in Heilongjiang province [16]. However, a big challenge for the management of SCN in the field is the presence of multiple SCN HG types [17]. In northeastern China, the majority of the resistant cultivars are derived from Peking via cultivar Franklin and/or its derived cultivars, whereas long-term, ongoing use of the same resistant source (Peking) caused the adaption of SCN populations in Heilongjiang [18].

Currently, HG type 7 is the most common SCN populations in Heilongjiang [19]. The predominant SCN virulence type of Jilin Province in Northeast China was also HG type 7 [20], while significant efforts have been made in Northeast China to develop soybean cultivars resistant to SCN HG type 0 [21]. In recent years, there has been an increase of SCN virulence, and a virulence shift of SCN populations has been reported in northeastern China [18,22]. HG type 1.3.4.7 were previously identified under continuous cropping in the Anda area of the Suihua region in Heilongjiang Province [16]. In addition, it is unclear which soybean cultivars are resistant to HG type 1.3.4.7. Once HG type 1.3.4.7 becomes a popular virulence phenotype, it will be a serious threat to soybean production. Although the reaction of soybean genotypes to SCN were reported in Northeast China [23], it was more important to test the response of soybean cultivar to the main or likely to be prevalent virulence phenotypes of SCN.

For the commercial soybean cultivars, no information is available on their effectiveness against the common HG type 7 and HG type 1.3.4.7. The objective of this research was to evaluate soybean cultivars for reaction to two nematode populations HG types 7 and 1.3.4.7 that are currently more prevalent or likely to be prevalent in the Heilongjiang than other populations to SCN.

## 2. Materials and Methods

### 2.1. Plant Materials

First, 110 domestic soybean cultivars including 25 SCN resistant soybean cultivars, 85 local high-yielding cultivars from Northeast China were evaluated for SCN resistance in the greenhouse. The 25 SCN resistant cultivars had SCN resistance to HG type 0. In addition, in these 25 SCN resistant soybean cultivars, 3 cultivars are from Jilin, and other 22 cultivars are all from Heilongjiang. Eighty-five representative commercial cultivars are from different ecological regions, such as a series of ‘Hefeng’, ‘Suinong’, ‘Dongnong’, ‘Heihe’, ‘Jiyu’, ‘Tiedou’, etc. (Table S1) These cultivars were obtained from Soybean Research Institute, Daqing Branch of the Heilongjiang Academy of Agricultural Sciences. PI 548402, PI 88788, PI 90763, PI 437654, PI 209332, PI 89772, PI 548316, Pickett, and the susceptible standard check Lee74 were also added to the test in order to confirm the virulence phenotypes of SCN [24].

### 2.2. Nematode Populations

The SCN populations included two near-homogeneous inbred lines that corresponded to HG types 7 and 1.3.4.7. The soil samples of HG types 7 and 1.3.4.7 were collected from soybean fields in Daqing region. These nematode populations had been maintained by Nematology Institute of Northern China of Shenyang Agricultural University for over 30 generations [25]. According to the previous inoculation method, the SCN was used for the following inoculation assays with 2000 eggs of SCN per plant [26]. There were five plants in each repetition, and the experiment was repeated twice. All plants were grown in greenhouses at 26–28 °C with an 16 h light/8 h darkness light cycle.

For inoculation assay, two seeds of each indicator were planted in pots (3.8 cm diameter and 14 cm length) with sandy loam soil (50% sand). After germination, one seedling was kept in each pot. Five replicated seedlings were used. At the second true leaf stage, each plant was inoculated with a 5.0 mL mixture suspension containing 2000 eggs of SCN. The pots were then arranged in a completely random experimental design in a greenhouse at 28 °C for 16 h light each day and were watered regularly. The soybean plants and soil were taken from the pots after 35 days and soaked in water for at least 30 min. Females were extracted from the roots and collected using an 80-μm-pore sieve [19].

### 2.3. Statistics

The female index was calculated as follows: FI = (mean number of females on test soybean line per mean number of females on Lee74) × 100. SCN populations were confirmed by HG Types classification schemes based on avirulence (FI < 10) or virulence (FI > 10) response [24]. The reaction levels for female index (FI) were R, resistant = 0–9%, MR, moderately resistant = 10–30%, MS, moderately susceptible = 31–60%, S, susceptible = >60% [27]. All the lines were included for statistical analysis. Data from two tests for HG types 7 and 1.3.4.7 were combined for the analysis of variance of the FIs by the SPSS statistics 26.0 (SPSS, Chicago, IL, USA). Means were separated using Fisher’s LSD based on a significant F test.

## 3. Results

### 3.1. Response of Indicator Lines to SCN Populations HG Types 7 and 1.3.4.7

The first population had FI > 10 on PI 548316 was confirmed as HG type 7. The second population had FI > 10 on PI 548402, PI 90763, PI 437654, and PI548316 was classified as HG type 1.3.4.7. According to race test, the first population had FI < 10 on PI 548402, PI 88788, PI 90763, Pickett was classified as race 3. and the second population had FI > 10 on Pickett was classified as race 14 (Table 1).

### 3.2. Resistance Response of Soybean Cultivars to SCN Populations HG Type 7

**Figure 1:**
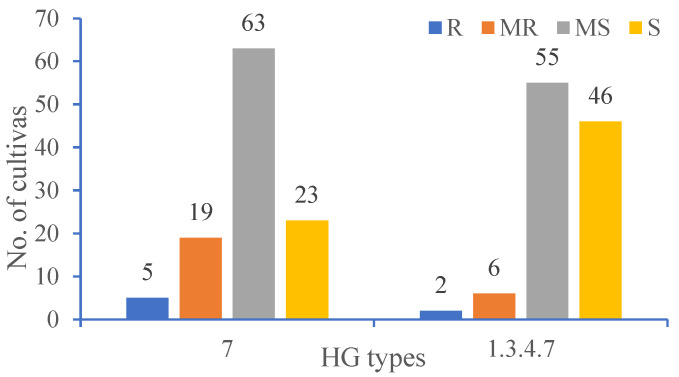
Frequency of cultivars assessed as R, resistant = 0–9%, MR, moderately resistant = 10–30%, MS, moderately susceptible = 31–60%, S, susceptible = >60% against SCN HG types 7 and 1.3.4.7.

Among 110 soybean cultivars, several cultivars with various levels of resistance to HG type 7 were shown in Table 1. There were five soybean cultivars displaying resistant (FI = 0–9%) to HG type 7, accounting for 4.55% of the total. Nineteen cultivars were moderately resistant (FI = 10–30%), accounting for 17.27% of the total. The number of HG type 7 with moderately susceptible (63) and susceptible (23) was 86 (Figure 1). Of these cultivars, the FI for the SCN populations ranged from 6.19 to 135.87. Five soybean cultivars from Heilongjiang, including Kangxian 2, Kangxian 5, Kangxian 7, Kangxian 12, and Qingdou 13 (also called Kangxian 13), showed resistance to SCN HG Type 7 (Table 2).

### 3.3. Resistance Response of Soybean Cultivars to SCN Populations HG Type 1.3.4.7

Two cultivars (Kangxian 12 and Qingdou 13) were resistant to HG type 1.3.4.7 (Race 14), accounting for 1.83% of the total. There were six cultivars with moderate resistance, accounting for 5.50% of the total number. The rest of the cultivars were moderately susceptible and susceptible (Figure 1). Among these cultivars, a minimum FI of 11.41 was evaluated for Kangxian 12 and a maximum FI of 141.93 was evaluated for Hefeng 25.

Among the 110 soybean cultivars, eight cultivars were resistant or moderately resistance to HG type 1.3.4.7. Six soybean cultivars showed moderate resistance to SCN HG type 1.3.4.7, including Bainong 9, Fengdou 3, Kangxian 10, Kangxian 7, Kangxian 6, and Kangxian 5. Bainong 9 is from Jilin, the other cultivars are from Heilongjiang. Kangxian 12 and Qingdou 13 showed resistance not only to SCN HG type 7 but also to SCN HG type 1.3.4.7 (Table 2).

### 3.4. Agronomic Characters of SCN Resistant Cultivars

Twenty-five soybean cultivars moderately resistant to HG type 7 have yellow seed coats. With regard to seed hilum color, almost 3/4 of the cultivars (72%, 18 out of 25) had brown-hilum-pigmented, the rest having either yellow or black seed hilum color. Six high-oil soybean cultivars, Dongnong 43, Nengfeng 18, Nengfeng 19, Kangxian 6, Pengdou 158 and Qinong 2, had the average contents of fat (oil) in the seeds more than 22%. The content of fat and protein in the seeds is one of the SCN-resistance breeding objectives. These cultivars have favorable agronomic characteristics, making them suitable for use as donor parents in SCN resistance breeding programs [19]. Twenty-two of the cultivars were cataloged as MG 0, whereas MG I had 3 sources. The hundred-seed weight and desirable agronomic traits of these cultivars were listed in Table 3. Agronomic traits were adopted from Qiu et al. and Lai et al. [28,29,30].

## 4. Discussion

This study evaluated the levels of resistance to two HG Types 7 and 1.3.4.7, in 110 commercial soybean cultivars from northeastern China. Kangxian 12 and Qingdou 13 showed resistance not only to SCN HG type 7 but also to SCN HG type 1.3.4.7. Kangxian 12 and Qingdou 13 had been reported to be resistant to HG type 2.5.7 and moderately resistant to both HG type 1.2.3.5.6.7, respectively [18]. Although some commercial cultivars were released as resistant to SCN type 0, the FI for these cultivars across the two HG types in our investigation was greater than 10% and 30%. Pickett was included in our study for the comparison of races between the two SCN populations so as to give soybean growers more information, although race and HG type belong to different classification systems.

In order to effectively manage SCN, it is crucial to understand the virulence phenotypes of SCN and resistant sources [31,32,33]. Soybean growers can select suitable resistant cultivars according to HG types and agronomic performance. In China, although a series of black-seed soybeans have multiple resistance to SCN, it is rarely used in commercial breeding programs due to lack of good agronomic traits [34]. SCN resistance was strongly associated with black seed coat. Soybean breeders need to make numerous backcrosses for improving undesirable traits related to the linkage of genetic background [17]. In this study, some cultivars which have yellow seed coat and good agronomic characteristics will be used in breeding programs as SCN resistance sources.

In previous studies, Kangxian 12 and Qingdou 13 were found to be resistant to HG types 2.5.7 and moderately resistant to HG type 1.2.3.5.6.7, respectively [18]. Here, we found that Kangxian 12 and Qingdou 13 has a broad spectrum resistance to SCN (HG Types 7, 1.2.5.7, HG types 2.5.7, and HG type 1.2.3.5.6.7). Furthermore, in order to create better soybean cultivars with resistance to SCN, the cultivar Kangxian 12 was used as a source of SCN resistance in soybean breeding programs. Some SCN resistant soybean cultivars, such as Nongqingdou 24 and Andou 162, were bred using Kangxian 12 as the male parent. Recently, a new cultivar ‘Heinong 531’ has been bred by means of systematic selection from the hybrids of Pengdou 158 × a male parental line F1 (Hefeng 55 × Kangxian 12) [21].

Kangxian12 were derived from the generations of the cross between Nongda 5129 and Heikang 002-24 which has SCN-resistance from Peking. Kangxian 12 carried resistant types of Forrest (*rhg1-a* GmSNAP18 and *Rhg4* GmSHMT08) [21]. In fact, most of the SCN-resistant soybeans were almost Peking-type in Heilongjiang province. However, in the USA, only a few SCN-resistant varieties come from Peking (PI 548402) and PI 437654, and most of the resistant soybean cultivars have the resistance gene of PI88788 in their pedigree.

Planting single soybean cultivar for many years may lead to the loss of yield due to the adaptation of SCN [35]. Broad-spectrum SCN resistance may be increased by stacking numerous sources of resistance [36]. Some resistant cultivars (Pengdou 158 and Qingdou 13) contain a complex genetic background. In the early soybean genetic improvement, many Chinese black beans and other resistance gene resources were aggregated into SCN resistant cultivars [37]. These include Huipizhiheidou, Wuzhaiheidou, Yingxianheidou, PI 548402, PI 437654, and PI 548316. In addition, PI 90763 and PI 209332 can also be used as sources of *H. glycines* resistance.

Although some cultivar resources are highly resistant to SCN, the majority of the soybean cultivar is susceptible to SCN. PI 437654 was identified as resistant to all SCN virulence types. However, the isolate TN27 could reproduce on PI 437654 were reported in USA, and the new virulence type (X12) could reproduce on all the indicator lines of both race and HG type tests in China [38,39]. The soybean cultivar from Northeast China breeding program was resistant to HG 0, whereas field populations of SCN exhibit variability in their parasitism of soybean cultivars. Thus, resistant varieties must be matched to the virulence phenotype of SCN [33].

Phenotyping identification is important for complicating the selection of resistant lines or the evaluation of management strategies [40]. In order to make better use of soybean cultivars with SCN resistance, virulence phenotype need to be carefully monitored in a manner similar to what is recommended by the SCN Coalition (www.thescncoalition.com). PI 88788 and PI 548402 with different ways of controlling SCN infection were used as a SCN resistant cultivar for many years. Thus, rotating different derived cultivars can reduce SCN density for sustainable management [41,42,43]. However, the yield of *H. glycines* resistant cultivars (Heinong 531 and Qingdou 13) is still lower than the local cultivars, soybean cultivars with excellent agronomic traits, and *H. glycines* resistance are still recommended in SCN infected fields. The source of SCN resistance in cultivated soybean gene pool is limited [44]. It may be a new strategy to identify SCN resistant gene resources from wild soybean to develop new cultivars [45].

## 5. Conclusions

This research provided important information on the reaction of 110 soybean cultivars to SCN populations HG Types 7 and 1.3.4.7. Of the local cultivars evaluated, five accessions were classified as resistant or moderately resistant to HG Type 7 and also displayed resistance to HG Type 1.3.4.7. Broad-based SCN resistance cultivars, Kangxian 12 and Qingdou 13, which had the resistance to the main or likely to be prevalent virulence phenotypes of *H. glycines*, would be valuable materials and can be used directly in the SCN resistance breeding program. Kangxian 12 with favorable agronomic characteristics is a valuable genetic reservoir for SCN resistance of soybean improvement. Our results provide guidance for the implementation of the strategy of using resistant cultivars to control SCN. The soybean germplasm collection and identification of SCN resistance are of paramount importance and will undoubtedly contribute to the development of different source against SCN.

## Figures and Tables

**Table 1:** Response of indicator lines to SCN populations HG Types 7 and 1.3.4.7.

HG Type	1 PI 548402 (Peking)	2 PI 88788	3 PI 90763	4 PI 437654	5 PI 209332	6 PI 89722	7 PI 548316 (Cloud)	Pickett
7	2.23	4.06	2.32	0	0	0	16.67	2.02
1.3.4.7	14.85	2.65	15.07	17.62	6.95	5.23	24.04	16.60

**Table 2:** Reaction of soybean cultivars to SCN HG types 7 and 1.3.4.7.

Cultivar	SCN HG Type 7			HG Type 1.3.4.7		
	Mean	FI %	Reaction	Mean	FI %	Reaction
Dongnong 43	50.60	22.69	MR	156.00	57.40	MS
Dongnong 44	147.80	66.28	S	143.25	52.70	MS
Dongnong 45	108.00	48.43	MS	244.00	89.77	S
Dongnong 46	78.50	35.20	MS	309.67	113.93	S
Dongnong 47	95.40	42.78	MS	106.75	39.28	MS
Dongnong 48	90.40	40.54	MS	116.40	42.83	MS
Dongnong 49	239.80	107.53	S	101.60	37.38	MS
Dongnong 50	128.20	57.49	MS	113.00	41.57	MS
Dongnong 51	170.00	76.23	S	101.40	37.31	MS
Dongnong 52	172.00	77.13	S	85.25	31.36	MS
Dongnong 61	103.60	46.46	MS	176.40	64.90	S
Dongnong 63	94.40	42.33	MS	121.00	44.52	MS
Nenfeng 14	37.40	16.77	MR	114.60	42.16	MS
Qinong 1	40.00	17.94	MR	94.67	34.83	MS
Nenfeng 16	77.20	34.62	MS	105.60	38.85	MS
Nenfeng 17	73.40	32.91	MS	91.80	33.77	MS
Nenfeng 18	22.33	10.01	MR	152.33	56.05	MS
Nenfeng 19	43.20	19.37	MR	117.00	43.05	MS
Nenfeng 20	26.00	11.66	MR	121.00	44.52	MS
Hefeng 25	303.00	135.87	S	265.50	97.68	S
Hefeng 30	250.00	112.11	S	109.00	40.10	MS
Hefeng 35	182.25	81.73	S	163.00	59.97	MS
Hefeng 55	94.20	42.24	MS	204.25	75.15	S
Hefeng 57	210.00	94.17	S	131.80	48.49	MS
Henong 58	163.40	73.27	S	284.75	104.76	S
Henong 59	227.20	101.88	S	250.00	91.98	S
Henong 60	122.40	54.89	MS	326.75	120.22	S
Hefeng 63	141.40	63.41	S	123.80	45.55	MS
Keshan 1	116.80	52.38	MS	103.75	38.17	MS
Beifeng 9	52.40	43.50	MS	126.60	46.58	MS
Beifeng 15	79.60	35.70	MS	101.20	37.23	MS
Beifeng 16	100.00	44.84	MS	110.60	40.69	MS
Beifeng 17	68.00	30.49	MS	106.60	39.22	MS
Fengshou 22	62.00	47.80	MS	106.60	39.22	MS
Fengshou 25	126.40	56.68	MS	265.00	97.50	S
Fengshou 26	115.00	51.57	MS	119.40	43.93	MS
Fengshou 29	40.60	18.21	MS	137.00	50.40	MS
Kangxian 2	15.40	6.91	R	93.40	34.36	MS
Kangxian 3	32.00	14.35	MR	127.00	46.73	MS
Kangxian 4	27.20	12.20	MR	101.20	37.23	MS
Kangxian 5	17.50	7.85	R	45.60	16.78	MR
Kangxian 6	30.40	13.63	MR	81.50	29.99	MR
Kangxian 7	19.40	8.70	R	61.00	22.44	MR
Kangxian 8	26.00	11.66	MR	104.50	38.45	MS
Kangxian 9	58.40	26.19	MR	98.20	36.13	MS
Kangxian 10	27.00	12.11	MR	79.50	29.25	MR
Kangxian 11	29.80	13.36	MR	157.00	57.76	MS
Kangxian 12	13.80	6.19	R	13.00	4.78	R
Qingdou 13	17.60	7.89	R	13.25	4.87	R
Fengdou 3	24.00	10.76	MR	63.00	23.18	MR
Pengdou 158	40.00	32.52	MR	101.00	37.16	MS
Bainong 9	40.25	32.72	MR	78.00	28.70	MR
Qinong 2	32.50	14.57	MR	134.33	49.42	MS
Suinong 37	40.20	18.03	MS	137.00	50.40	MS
Suinong 39	24.60	11.03	MS	114.40	42.09	MS
Kennong 21	126.80	56.86	MS	131.50	48.38	MS
Kennong 20	81.75	36.66	MS	132.67	48.81	MS
Kennong 19	184.00	82.51	S	171.33	63.04	S
Kennong 18	94.20	42.24	MS	119.60	44.00	MS
Kennong 17	113.60	50.94	MS	148.25	54.54	MS
Kennong 16	100.00	47.62	MS	164.50	85.68	S
Kenfeng 16	138.00	65.71	MS	104.20	54.27	MS
Kenfeng 15	138.80	66.10	MS	169.25	88.15	S
Kenfeng 13	76.75	36.55	MS	169.50	88.28	S
Kenfeng 12	102.80	48.95	MS	125.25	65.23	S
Kenfeng 11	141.60	67.43	MS	94.80	49.38	MS
Kenfeng 10	93.40	44.48	MS	87.00	45.31	MS
Heinong 41	202.80	96.57	S	111.20	57.92	MS
Heinong 48	109.20	52.00	MS	119.80	62.40	S
Heinong 47	110.00	52.38	MS	105.80	55.10	MS
Heinong45	149.25	71.07	S	130.00	67.71	S
Heinong 54	95.75	45.60	MS	103.40	53.85	MS
Heinong 43	97.00	43.50	MS	107.80	56.15	MS
Heinong 58	153.00	72.86	MS	135.60	70.63	S
Heinong 60	78.40	35.16	MS	126.20	65.73	S
Heinong 63	121.00	54.26	MS	117.40	61.15	S
Heihe 42	116.50	55.48	MS	136.80	71.25	S
Heihe 41	76.80	36.57	MS	131.33	68.40	S
Heihe 40	105.00	50.00	MS	189.00	98.44	S
Heihe 39	118.75	56.55	MS	170.67	88.89	S
Heihe 38	91.00	43.33	MS	223.50	116.41	S
Heihe 37	111.25	52.98	MS	113.60	59.17	MS
Heihe 35	83.20	39.62	MS	123.67	64.41	S
Heihe 34	68.00	32.38	MS	109.50	57.03	MS
Heihe 33	83.25	39.64	MS	125.00	65.10	S
Heihe 43	96.00	45.71	MS	109.75	57.16	MS
Bainong 5	40.60	19.33	MR	91.40	47.60	MS
Bainong 6	85.40	40.67	MS	176.00	91.67	S
Bainong 8	32.00	15.24	MR	153.00	79.69	S
Dongsheng 1	76.50	36.43	MS	192.00	100.00	S
Dongsheng 2	170.00	80.95	S	206.50	107.55	S
Dongsheng 7	70.40	33.52	MS	201.00	104.69	S
Dongsheng 3	180.60	86.00	S	174.50	90.89	S
Dongsheng 9	83.80	39.90	MS	153.67	80.03	S
Jiyu 77	75.20	35.81	MS	91.33	47.57	MS
Jiyu 99	124.60	59.33	MS	121.50	63.28	S
Jiyu 303	96.00	45.71	MS	167.50	87.24	S
Jiyu 403	105.00	50.00	MS	181.33	94.44	S
Jiyu 47	103.20	49.14	MS	272.50	141.93	S
Jiyu 86	129.00	61.43	S	195.80	101.98	S
Jidadou 3	101.20	48.19	MS	202.60	105.52	S
Jidadou 5	199.00	94.76	S	140.80	73.33	S
Liaodou 15	182.50	86.90	S	107.80	56.15	MS
Liaodou 28	135.80	64.67	S	143.00	74.48	S
Liaodou 32	93.60	44.57	MS	124.40	64.79	S
Tiedou 53	177.00	84.29	S	147.80	76.98	S
Tiedou 63	98.00	46.67	MS	N/A	N/A	N/A
Tiedou 71	108.40	51.62	MS	192.00	100.00	S
Tiedou 72	146.60	69.81	S	170.40	88.75	S
Tiedou 73	260.50	124.05	S	118.20	61.56	S
CV (%)		34.52			22.43	
LSD_0.05_		19.20			10.06	

Note: mean = mean number of females occurring on the soybean lines, N/A = No data. The reaction levels for female index (FI) were R, resistant = 0–9%, MR, moderately resistant = 10–30%, MS, moderately susceptible = 31–60%, S, susceptible = >60%.

**Table 3:** Soybean cultivars with SCN resistance having desirable agronomic characteristics.

Cultivar	Province	HG Type 7		HG Type 1.3.4.7		Pedigree	Protein Content/%	Fat Content/%	Maturity Group	Seed Color	Hilum Color	Height	100-Seed Weight
Dongnong 43	Heilongjiang	22.69	MR	57.40	MS	Suinong 8 × CN 210	40.21	22.97	0	Y	Br	90	20
Nengfeng 14	Heilongjiang	16.77	MR	42.16	MS	An 70-4176	43.98	19.70	0	Y	Br	95	22
Qinong 1	Heilongjiang	17.94	MR	34.83	MS	(Nen 950127-4 × Dongnong 42) × Nenfeng 16	40.46	21.53	0	Y	Br	100	22
Nenfeng 18	Heilongjiang	10.01	MR	56.05	MS	Nen92046 F1 × Hefeng 25	38.22	22.69	0	Y	Br	90	20
Nenfeng 19	Heilongjiang	19.37	MR	43.05	MS	Nen 76569-17 × 334 Mutagenic Offspring	37.86	22.05	0	Y	Br	90	18
Nenfeng 20	Heilongjiang	11.66	MR	44.52	MS	Hefeng 25 × An 8711-277	41.72	19.82	0	Y	Br	88	22
Kangxian 2	Heilongjiang	6.91	R	34.36	MS	Nenfeng 9 × (Nenfeng 10 × Franklin) F2	37.10	20.31	0	Y	Br	95	18
Kangxian 3	Heilongjiang	14.35	MR	46.73	MS	8201-205 × 8314-1222	37.77	21.77	0	Y	Y	95	20
Kangxian 4	Heilongjiang	12.20	MR	37.23	MS	8108-5 × Jiufeng 1	38.20	20.77	0	Y	Br	70	20
Kangxian 5	Heilongjiang	7.85	R	16.78	MR	Hefeng 25 × 8804-33	41.18	19.75	0	Y	Br	80	20
Kangxian 6	Heilongjiang	13.63	MR	29.99	MR	An 8201-205 × D-Haidou	38.17	22.06	0	Y	Br	90	20
Kangxian 7	Heilongjiang	8.70	R	22.44	MR	Hefeng 36 × Kangxian3	38.97	19.98	0	Y	Br	95	20
Kangxian 8	Heilongjiang	11.66	MR	38.45	MS	An 95-1409 × Donong xiaolidou 690	40.10	20.37	0	Y	Br	85	21
Kangxian 9	Heilongjiang	26.19	MR	36.13	MS	Heinong 37 × An 95-1409	40.09	21.22	0	Y	Br	85	20
Kangxian 10	Heilongjiang	12.11	MR	29.25	MR	Hefeng 33 × Kangxian 3	42.30	19.22	0	Y	Br	85	21
Kangxian 11	Heilongjiang	7.89	R	57.76	MS	Dongnong 434 × (An 01-1767 × An 87-7163) F1	39.41	21.50	0	Y	Bl	85	21
Kangxian 12	Heilongjiang	6.19	R	4.78	R	Heikang 002-24 × Nongda 5129	39.77	20.89	0	Y	Bl	90	19
Qingdou 13	Heilongjiang	7.89	MR	4.87	R	Heikang 002-24 × Nongda 5129	41.06	21.09	0	Y	Bl	90	19
Fengdou 3	Heilongjiang	10.76	MR	23.18	MR	Kangxian 4 × Suinong 14	39.61	21.22	0	Y	Br	80	22
Pengdou 158	Heilongjiang	17.94	MR	37.16	MS	(Dongnong 46 × 9902) F1 × Nongda 5129	39.08	22.16	0	Y	Br	80	22
Qinong 2	Heilongjiang	14.57	MR	49.42	MS	Ha 4475 × Nenfeng 17	38.23	21.48	0	Y	Br	114	18
Bainong 9	Jilin	18.05	MR	28.70	MR	Baijiao 8209-8 × Jilin 20	39.88	22.29	I	Y	Br	95	18
Bainong 5	Jilin	19.33	MR	47.60	MS	(Jiti 5 × Silihuang) F2 × Qunxuan 1	41.40	20.07	I	Y	Y	90	17
Bainong 8	Jilin	15.24	MR	79.69	S	(Changnong 4 × Jilin 20) F1× Jilin 27	40.45	19.58	I	Y	Y	95	20

Note: Y = yellow, Bl = black, Gn = green, Br = brown.

## Data Availability

The original contributions presented in the study are included in the article. Further inquiries can be directed to the corresponding author/s.

## Supplementary Materials

The following supporting information can be downloaded at: https://www.mdpi.com/article/10.3390/life13010248/s1. Table S1. List of soybean cultivars evaluated in previous study.
