# Supplementary material for: Evaluation Soybean Cultivars for Reaction to Heterodera glycines Populations HG Types 7 and 1.3.4.7 in Northeast China
Source: Life (Basel). 2023 Jan 16;13(1):248. doi: 10.3390/life13010248 (PMC9864252; doi:10.3390/life13010248)
Supplement: Supplementary file 1 [file life-13-00248-s001.zip › life-2124025-supplementary.pdf]

Supplementary Table S1. List of soybean cultivars evaluated in previous study.

Genotype Category	Province	Soybean Cultivars
SCN-resistant cultivars （HG 0）	Heilongjiang	Kangxian 2，Kangxian 3，Kangxian 4，Kangxian 5，Kangxian 6，Kangxian 7，Kangxian 8，Kangxian 9，Kangxian10，Kangxian 11，Kangxian 12，Qingdou 13，Nenfeng 14，Nenfeng 17，Nenfeng 18，Nenfeng 19，Nenfeng 20 ，Qinong 1，Qinong 2，Dongnong 43，Fengdou 3，Pengdou 158
	Jilin	Bainong 9，Bainong 5，Bainong 8
Commercial cultivars	Heilongjiang	Dongnong 44，Dongnong 45，Dongnong 46，Dongnong 47，Dongnong 48，Dongnong 49，Dongnong 50，Dongnong 51，Dongnong 52，Dongnong 61，Dongnong 63，Hefeng 25，Hefeng 30，Hefeng 35，Hefeng 55，Hefeng 57，Henong 58，Henong 59，Henong 60，Hefeng 63，Beifeng 9，Beifeng 15，Beifeng 16，Beifeng 17，Keshan 1，Fengshou 22，Fengshou 25，Fengshou 26，Fengshou 29， Suinong 37，Suinong 39，Kennong 21，Kennong 20，Kennong 19，Kennong 18，Kennong 17，Kennong 16，Kenfeng 16，Kenfeng 15，Kenfeng 13，Kenfeng 12，Kenfeng 11，Kenfeng 10，Heinong 41，Heinong 48，Heinong 47，Heinong 45，Heinong 54，Heinong 43，Heinong 58，Heinong 60，Heinong 63，Heihe 42，Heihe 41，Heihe 40，Heihe 39，Heihe 38，Heihe 37，Heihe 35，Heihe 34，Heihe 33，Heihe 43，Dongsheng 1，Dongsheng 2，Dongsheng 7，Dongsheng 3，Dongsheng 9，Jidadou 3，Jidadou 5，Nenfeng 16
	Jilin	Jiyu 77，Jiyu 99，Jiyu 303，Jiyu 403，Jiyu 47，Jiyu 86，Bainong 6
	Liaoning	Liaodou 15，Liaodou 28，Liaodou 32，Tiedou 53，Tiedou 63，Tiedou 71，Tiedou 72，Tiedou 73
